# Determination of alpha factors for monitoring of aeration systems with the ex situ off-gas method: experience from practical application and estimation of measurement uncertainty

**DOI:** 10.1007/s11356-022-21915-2

**Published:** 2022-07-12

**Authors:** Maximilian Schwarz, Jana Trippel, Markus Engelhart, Martin Wagner

**Affiliations:** Institute IWAR, Chair of Wastewater Technology, Technical University of Darmstadt, Franziska-Braun-Str. 7, 64287 Darmstadt, Germany

**Keywords:** Activated sludge, Aeration, α-Factor, Measurement uncertainty, Oxygen transfer, Sensitivity analysis

## Abstract

Performance of aeration systems in wastewater treatment plants (WWTP) under process conditions can be monitored with off-gas tests. The ex situ off-gas method transfers activated sludge from an adjacent aeration tank into aerated columns to determine oxygen transfer parameters (e.g., the α-factor). This method is an alternative to in situ off-gas testing with hoods at the tank surface; however, its application and measurement uncertainty have not been examined yet. We outline our experience from long-term off-gas testing with two pilot-scale test reactors (8.3 m^3^ volume). Global variance-based sensitivity analysis using Sobol’ indices revealed oxygen concentration in off-gas and dissolved oxygen as the most important input quantities to determine α-factors accurately. Measurement uncertainty of other instruments was negligible. These findings are transferable to in situ off-gas hoods because the methods are similar. Random measurement error of α-factors was estimated with uncertainty analysis and comparison measurements to a relative standard deviation of about ± 2.8% for our ex situ pilot setup. Diffuser fouling, biofilm growth, or sensor drift caused systematic errors avoidable by maintenance. Additional mixing of bubble column due to sludge inflow into ex situ tanks led to a systematic overestimation of α-factors at lower airflow rates. Hence, the ex situ off-gas method is not suitable to determine α-factors for the design of aeration systems but offers unique possibilities for research of oxygen transfer dynamics and development of aeration equipment because ex situ columns can be operated independently from a full-scale activated sludge tank.

## Introduction

Aeration is an energy-intensive process in activated sludge (AS) biological wastewater treatment. Measurement of oxygen transfer parameters in activated sludge tanks is essential for design and operation of aeration systems. Clean water testing is an established method to determine oxygen transfer performance of diffusers (ASCE 2–06 2007; EN 12,255–15 2003). Still, wastewater treatment plant (WWTP) operators face a decline of oxygen transfer under process conditions in activated sludge tanks. This is caused by inhibitory effects of wastewater and activated sludge components in the soluble and solid phase as well as the impact of fouling, scaling, and aging of diffusers resulting in poor bubble formation and rise as reviewed by Baquero-Rodríguez et al. (2018). The α-factor summarizes these oxygen transfer inhibiting effects as the ratio of oxygen transfer in process water to clean water. Design and operation of aeration systems must consider oxygen transfer in process conditions, which can be measured with off-gas methods (ASCE 18–18, 2018; DWA-M 209 2007).

### Off-gas testing in wastewater treatment

Off-gas testing methods have been used for numerous applications in design and operation of aeration systems as well as research of gas transfer in activated sludge, as the following examples show. In several studies, off-gas tests were used to examine impacts on oxygen transfer by activated sludge characteristics or WWTP operation and process layout (Leu et al. 2009; Rosso et al. 2008, 2005; Schuchardt et al. 2005). Studies of this type allow to model aspects of the oxygen transfer. For example, Jiang et al. (2017) proposed a dynamic model to predict α-factors based on the relationship between the α-factor and chemical oxygen demand (COD). Off-gas tests can be part of the design process of the aeration system. Rosso et al. (2012) performed on-site testing of various diffusers to determine the influence of process specific wastewater properties on oxygen transfer (and pressure loss) during design phase to enable more accurate design of aeration systems. Off-gas tests can also be utilized to monitor the operation of a WWTP. Trillo et al. (2004) applied off-gas hoods for a feed-forward dissolved oxygen (DO) control to reduce aeration energy costs. Leu et al. (2010) measured oxygen and carbon dioxide transfer rates to predict effluent ammonia. Hellinga et al. (1996) already argued that in contrast to selective point measurements with sensors, off-gas measurements in treatment plants with covered aeration tanks could be a worthwhile addition to liquid phase analysis to monitor the overall biological treatment process. This application of off-gas testing could also be combined with monitoring of emissions in the future. Myers et al. (2021) measured dissolved and off-gas nitrous oxide (N_2_O) in a conventional activated sludge (CAS) WWTP and estimated volumetric mass transfer coefficient of nitrous oxide based on the mass transfer coefficient for oxygen. Baeten et al. (2021) used off-gas analysis to detect several emissions (CO_2_, CH_4_, N_2_O) in an aerobic granular sludge WWTP. So far, off-gas analyses have been used in numerous studies, but application of gas analyzers is not part of the typical instrumentation on WWTPs yet.

### Comparison of off-gas methods

The off-gas method with off-gas hoods on the surface of aeration tanks is first described by Redmon et al. (1983) and explained in ASCE/EWRI 18–18 (2018) and DWA-M 209 (2007). It allows to measure oxygen transfer efficiency from which an oxygen uptake rate (OUR) can be calculated based on a dissolved oxygen mass balance. Boyle et al. (1989) demonstrate the possibilities of off-gas measurements for OUR online monitoring without the necessity of error-prone ex situ batch OUR respirometry devices. The Redmon Engineering Company used ex situ off-gas column tests to determine α-factors in the 1980s. The method is first described by Rieth and Polta (1987) and included in ASCE 18–18, section D.1.4.4. We refer to the method as ex situ column off-gas testing. It is an alternative to in situ off-gas hoods that allows to examine oxygen transfer in activated sludge transferred from an adjacent AS tank into a separate column. The off-gas measurement is therefore independent from the operation of the activated sludge tank and its aeration system. Both the in situ off-gas hood and the ex situ column method allow to determine the same oxygen transfer parameters, e.g., standard oxygen transfer rate (SOTR), standard oxygen transfer efficiency (SOTE), OUR, and the α-factor. However, the application differs in certain aspects of the methodology and operation.

Placing multiple off-gas hoods to cover an activated sludge tank is generally less expensive than using ex situ columns to reach the same coverage. Determination of an overall α-factor of the process design is especially relevant in plug-flow reactors, tapered aeration or tanks with varying oxygen concentrations in different tank areas (Rosso et al. 2005; Stenstrom et al. 2006). Additionally, more sensors and flow measurements are necessary with the ex situ off-gas measurement compared to off-gas hoods which increases maintenance effort. In situ off-gas hoods are more convenient for a WWTP operator to monitor an installed aeration system’s performance over long periods or estimate it with single off-gas test series. However, a variation of the oxygen transfer cannot be attributed distinctly to either activated sludge related (*α*) or fouling related (*F*) causes. In contrast, ex situ columns allow to mitigate fouling by regular cleaning of diffusers and therefore distinguish the α-factor from the fouling factor. In addition, clean water testing is mandatory to determine the α-factor and easier to perform in ex situ columns than in a full-scale AS tank. Overall, an ex situ column allows to change certain properties of the aeration system and operation without interfering with the WWTP operation. Applications for research purposes could include varying tank geometry (especially blow-in depth), changing diffusers to find suitable types for a certain application, examining the effect of maintenance methods (e.g., reverse flexing, high-pressure cleaning, and acid injection) on reinstating pressure loss of diffusers, or performing spiking experiments to change wastewater characteristics and study the resulting effect on oxygen transfer. While off-gas hoods can only be placed in aerated zones, the ex situ method allows to transfer sludge from non-aerated zones into the column and determine oxygen transfer parameters. This also allows to determine oxygen transfer parameters for activated sludge in tanks without submerged aeration systems or covered tanks where hoses for sludge transfer can be installed unlike off-gas hoods. This represents a unique advantage of the ex situ off-gas method to research potential emissions from non-aerated zones in the future.

### Estimation of measurement uncertainty

Technical guidelines such as ASCE 18–18 or DWA-M 209 define measurement models to determine oxygen transfer parameters, e.g., oxygen transfer rate (OTR), oxygen transfer efficiency (OTE), or the α-factor. These measurement models define a functional relationship between several input quantities recorded by sensors or instruments and an output quantity, e.g., the α-factor as a measurand. Currently, guidelines are missing a stochastic component that considers measurement uncertainty of instruments recording input quantities. Instead, guidelines propose a measurement uncertainty to be expected for results if the applied method was conducted according to standard. For the use of in situ off-gas hoods DWA-M 209 (2007) estimated a measurement uncertainty of ± 5 to 10% for SOTR in activated sludge depending on tank shape and size. ASCE 18–18 (2018) referred to a comparison of several methods by Capela et al. (2004) and Mahendraker et al. (2005) and concluded that the examined methods estimated oxygen transfer parameters in activated sludge within 10 to 15% of each other depending on the examined method. Redmon et al. (1983) originally reported a reproducibility of ± 10% for OTE with in situ off-gas tests in activated sludge which primarily depended on changing conditions at a sampling point rather than accuracy of the analytical system.

None of the studies examined the ex situ column off-gas method. In addition, it remains unclear which measured input quantity is most important when determining oxygen transfer parameters or the α-factor. Another detail that is often accepted without further revision is the use of correction terms for standardization. For off-gas methods β and θ correction factors are applied to consider the effect of salts on the effective oxygen saturation and temperature on the oxygen transfer, respectively. These empirically determined correction terms are also estimates of quantities which are known imperfectly and could vary between applications of off-gas tests (Stenstrom and Gilbert 1981). Sensitivity analysis is a method to examine these issues. Its principle is to identify the effect of changes or uncertainty of input quantities on the model output (Turányi 1990). Variance-based methods such as Sobol’s method (Sobol’ 1993) aim to explain the effect of variance in model inputs on variance in model outputs. The thereby calculated sensitivity indices distinguish first-order and total effects indices. A first-order index represents the influence of an individual input quantity on the variance of the model output quantity. A second-order index explains interactions between two input quantities on the model output which cannot be explained by the sum of their first-order effects. Total effects indices summarize all higher-order indices (including second-order and above) to represent the total impact of an input quantity on output variance (Homma and Saltelli 1996). Sensitivity indices are represented by values between 0 and 1. When comparing sensitivity indices of input parameters, a higher value indicates a stronger influence of the input quantity on the model output. It is therefore more important to define or measure accurately to yield reasonable results. The methodology of sensitivity analysis (SA) using Sobol’ indices is described, e.g., in Saltelli et al. (2004) or Sobol’ and Kucherenko (2005).

## Objectives

Our study describes the setup and operation of the ex situ column off-gas method in-depth and thereby complements information missing in technical standards. In addition, there are three objectives to improve future applications of the ex situ column off-gas method: (1) we determine the most influential input quantities for determination of α-factors according to ASCE 18–18 with a sensitivity analysis, (2) we estimate the method’s random and systematic measurement error, and (3) we discuss causes of these errors and other constraints of the ex situ column off-gas method.

## Methods

### *Design and operation of *ex situ* columns*

**Fig. 1:**
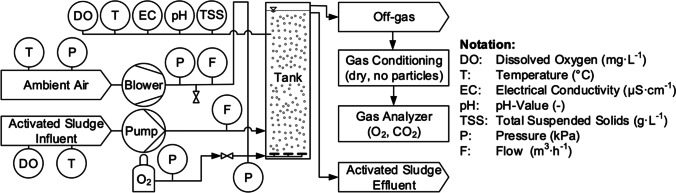
Flow diagram of an ex situ setup for steady-state off-gas measurements

Pilot-scale test reactors were used to determine oxygen transfer parameters applying an off-gas method described in Appendix D.1.4.4 of ASCE/EWRI 18–18 (2018). The method is a variant of the steady-state oxygen uptake rate (OUR) technique, where OUR is measured within the ex situ columns with off-gas analysis instead of an additional respirometry device. Figure 1 shows a flow diagram of the process for one ex situ aeration tank. Our pilot plant featured two aeration tanks with duplicate machinery and instruments to examine two AS tank zones in parallel.

Tank dimensions were 1.2 m × 1.2 m × 5.8 m (L × W × H) with a volume of 8.3 m^3^. The tank height was chosen to resemble typical water depths of AS tanks and therefore bubble rise conditions in the column. Columns were equipped with fine-bubble disc diffusers with a diffuser density of 13.5% (ELASTOX-T EPDM TYP B, WILO GVA, Wülfrath, Germany). Oil-free rotary vane vacuum blowers (CB.40, D.V.P. Vacuum Technology spa, Italy) were controlled by frequency converters to set airflow rates (specified for aerated tank volume − *q*_Vol,aer_) between 0.5 and 2.5 Nm^3^∙m^−3^∙h^−1^. Airflow rate was standardized (101.325 kPa, air temperature of 0 °C, dry air) and measured with thermal mass flowmeters (t-mass 150, Endress + Hauser AG, Reinach, Switzerland) in the inflow only. As with in situ off-gas hoods, this assumes that inert gas constituents such as nitrogen are conservative within the reactor and therefore net transfer of these gases is negligible. Pressure in air pipes (Cerabar PMC21, Endress + Hauser AG, Reinach, Switzerland) was measured after blowers and before diffuser distribution frame to determine pipe pressure loss and diffuser pressure loss. Sludge transfer pumps (AGNM02 NEMO®, NETZSCH Holding, Selb, Germany) pumped AS from a nearby AS tank through DN 100 hoses into the columns at the height of the disc diffusers. Depending on the examined AS tank zone a hose length of up to 100 m was installed and the maximum transfer time of the AS to the test columns was 90 s. Sludge flow was measured with electromagnetic flowmeters (Promag W 400, Endress + Hauser AG, Reinach, Switzerland) and transfer pumps controlled by frequency converters to set a constant hydraulic retention time (HRT) of 15 min as recommended by ASCE/EWRI 18–18. Effluent sludge was directed in free flow through a DN 150 hose from an overflow edge back into the nearby AS tank downstream of sludge intake.

Determining oxygen transfer parameters of AS in the columns required further sensors and instruments for measurement. Atmospheric pressure (Cerabar PMC21, Endress + Hauser AG, Reinach, Switzerland), atmospheric temperature (Omnigrad T TST434, Endress + Hauser AG, Reinach, Switzerland), and electrical conductivity in AS (Indumax CLS50D, Endress + Hauser AG, Reinach, Switzerland) were measured for standardization of oxygen transfer parameters (20 °C water temperature, 101.325 kPa atmospheric pressure, 1.000 mg·L^−1^ total dissolved solids). Off-gas concentrations of oxygen (paramagnetic sensor) and carbon dioxide (NDIR) were recorded with a gas analyzer (X-STREAM Enhanced, Emerson Electric Co., MO, USA) that received dry off-gas free of particles from a gas conditioning unit (CSS-V, M&C TechGroup, Ratingen, Germany). Off-gas was collected from the sealed column hood. To quickly monitor changing process conditions, a low hood height of 0.2 m above water surface was implemented on top of the columns. Depending on airflow rate setting the mean gas sample residence time in the hood was between 2 and 4 min, which included off-gas transport from hood to gas analyzer. Foaming could complicate off-gas collection in low hoods. Thus, the pilot plant was equipped with a U-shaped off-gas pipe that withheld foam from off-gas collection.

Sensors were cleaned twice a week to prohibit biofilm growth and solids deposition affecting optical instruments and calibrated as necessary. Because of its relevance for the off-gas method, two-point calibration of the off-gas analyzer was performed twice a week using calibration gases with 5% CO_2_, 16% O_2_, and 100% N_2_ for zero point. Potential biofilm build-up on the reactor tank walls was prevented with monthly cleaning and visual inspection to ensure only suspended biomass transferred from the adjacent full-scale AS tanks was examined in the ex situ reactors for off-gas measurements.

### Data recording and processing

A suitable interval for data compression must be short enough to record changes in WWTP operation or wastewater composition that could affect oxygen transfer and long enough to produce distinguishable datapoints for further analysis. Depending on the response time of equipped sensors in a pilot plant, determining α-factors in intervals of a few minutes is possible. A typical measurement period for off-gas testing is 30 min to 2 h (ASCE 2018). From the recorded data, a mean α-factor and a dispersion coefficient (e.g., standard deviation) is determined to estimate uncertainty of measurement or steady-state conditions. In continuous ex situ measurements these α-factors form a time series that describes the change of oxygen transfer in the continuous stirred tank reactor (CSTR). However, the determined oxygen transfer parameter or α-factor does represent not only the oxygen transfer of the sludge inflow at that moment but also of the previously transferred activated sludge already in the column. Therefore, determination of α-factors in an ex situ CSTR requires longer intervals depending on hydraulic retention time (HRT) and the resulting residence time distribution of the activated sludge in the columns. From our experience sufficient mixing was provided by aeration in the columns. An airflow rate of 2.2 Nm^3^·m^−2^·h^−1^ (0.38 Nm^3^·m^−3^·h^−1^), which is a commonly used design criterion to maintain solids in suspension (Water Environment Federation, 2018), was exceeded during off-gas testing. Additionally, a constant lateral flow of activated sludge transferred into the tank potentially mixed dead space beneath the diffuser distribution frame. Unless a sensor drift occurred, DO sensors showed the same DO concentration in the reactors. Therefore, ideal mixing conditions within the columns can be assumed and the residence time distribution (*t*) in a single ideal CSTR can be expressed as a cumulative distribution function as

1 $${F}_{(t)}=1-{e}^{-\frac{t}{\mathrm{HRT}}}$$

Based on this ideal relationship, in our pilot plant, 63% of activated sludge transferred into the test column was exchanged within the HRT of 15 min. Accordingly, after 30, 45, and 60 min, 86%, 95%, and 98% of sludge were replaced. As a result, a 1-h interval is a suitable interval for data compression for an ex situ reactor operated at an HRT of 15 min to determine α-factors.

To maintain steady-state conditions within a selected interval of data compression, some parameters (i.e., reactor influent flow, influent DO, DO in reactor, oxygen uptake rate, and oxygen transfer rate) should remain constant to determine oxygen transfer parameters (Boyle 1983). Therefore, ex situ columns allow to control reactor inflow and internal DO. Influent DO is steady if the examined activated sludge tank is controlled to a DO setpoint. However, oxygen uptake rate and oxygen transfer rate depend on wastewater composition and operation of AS process. Both are variable throughout a longer measurement period. Consequently, a test period to determine α-factors must be long enough to collect data sufficiently and short enough to keep steady-state conditions.

In our setup, data was recorded in 30-s intervals by online sensors and compressed as 1-h averages. This results in high resolution data that can detect variations within the diurnal cycle of WWTP operation. It also prevents autocorrelation of measured values and converts the collected time series data to resemble a cross-sectional dataset. From our experience, the required constant conditions as described above were met within a 1-h interval unless airflow rate or DO setpoint were changed manually or according to a schedule within an interval.

### Determination of oxygen transfer parameters

Determination of the α-factor and other oxygen transfer parameters is based on the well-established equation for actual oxygen transfer rate under process conditions (AOTR) which represents the transfer of oxygen without any standardization in activated sludge (United States Environmental Protection Agency 1989). The equation contains several factors to consider the influence of wastewater characteristics and varying ambient conditions during off-gas testing. Rearranged for the α-factor it is expressed as:

2 $$\alpha =\frac{\mathrm{AOTR}}{F\cdot {k}_{L}{a}_{cw,20}\cdot (\beta \cdot \tau \cdot \Omega \cdot {C}_{20}^{*}-{C}_{\left(t\right)})\cdot {\theta }^{T-20}\cdot V}$$

In off-gas measurements, AOTR (g·h^−1^) is calculated from the oxygen transfer efficiency (OTE) at a certain airflow rate. ASCE 18–18 describes how OTE is calculated from a mass balance of inlet and outlet oxygen and carbon dioxide concentrations measured with an off-gas analyzer. It also defines dimensionless standardization parameters to calculate standard oxygen transfer rate (SOTR) where τ is the oxygen saturation ratio at operating temperature and at 20 °C, *Ω* is the oxygen saturation pressure correction factor for 101.325 kPa, *β* is the ratio of oxygen saturation in process water and clean water, and *θ* is the temperature correction coefficient for water temperatures of 20 °C. Although ASCE 18–18 provides a general description of the ex situ column method, we provide further explanations based on practical experience below and added all equations to determine the α-factor in Appendix 1 of this paper.

#### Fouling factor —F ( −)

The fouling factor is defined as the ratio of oxygen transfer performance of used and new diffusers. During long-term off-gas measurements in activated sludge, diffuser performance is reduced because of scaling, fouling, and aging of diffusers. Ex situ columns could be used to specifically determine the fouling factor *F* if diffusers were not cleaned periodically. Significant increases of fouling measured by pressure loss are rare within the first three months without maintenance (Rosso 2015; Rosso et al. 2012). On the other hand, ex situ columns allow to maintain diffuser performance and therefore to determine α-factors with minimal impact of fouling if maintained properly within shorter intervals. To mitigate fouling, regular pressure cleaning and reverse flexing of diffusers and acid addition into air pipes can be performed (Odize et al. 2017; Rosso 2018; Wagner and Stenstrom 2014). Because our objective was to determine oxygen transfer as α-factor instead of αF-factor, reverse flexing was performed twice a week and membrane surface of diffusers was cleaned with high pressure once a month. A previous study has shown that the effect of fouling during long-term off-gas measurements could be kept low when applying this maintenance (Schwarz et al. 2021). Here, clean water tests repeated over a period of 13 months revealed a decrease of SOTR of 2 to 6% depending on airflow rate and a dynamic wet pressure increase of about 1 kPa. For even longer periods, an exchange of diffusers seems advisable.

#### ***Clean water testing— k***_***L***_***a***_***cw,20***_

Clean water (cw) testing is required to determine the denominator of the α-factor which is based on the linear relationship between airflow rate and SOTR in clean water. We used different probes in clean water and process water because clean water tests required faster dissolved oxygen (DO) probes than off-gas measurements at high airflow rates. Electrochemical DO probes (Oxymax COS51D, Endress + Hauser AG, Reinach, Switzerland) with a fast response time *t*_90_ of 30 s were used. Slower optical DO probes Oxymax COS61D, Endress + Hauser AG, Reinach, Switzerland) produced similar results but at lower accuracy. These were used in process conditions as long-term testing did not require a fast response time and their lower maintenance allowed more reliable operation in activated sludge. Furthermore, off-gas measurements were performed at a steady sludge inflow, while non-steady-state clean water tests were not. Consequently, differences of bubble rise and gas holdup in the columns could have occurred between test methods as discussed later. A steady-state clean water test is neither described in technical guidelines nor practically feasible at the setup’s scale.

#### Oxygen saturation concentration—C^*^_20_

Steady-state off-gas cannot provide an estimate of effective oxygen saturation concentration C^*^_20_ which would result in the activated sludge at zero respiration rate. Therefore, it was estimated by a mid-depth model also considering influence of temperature and pressure (i.e., *τ*, *Ω*) (compare with Jiang and Stenstrom 2012). However, the effect of soluble total dissolved solids (TDS) as estimated by the β-factor cannot be determined in continuous off-gas testing. Instead, it is estimated from electrical conductivity by a conversion factor of 2 mg·L^−1^ TDS/3 µS·cm^−1^ (see DWA-M 209, 2007).

#### Volume V

Volume of tanks should be measured accurately because it directly affects SOTR. Clean water and off-gas testing should be conducted with the same water volume to prevent a systematic error.

#### α-Factor

To determine the α-factor in the aeration tank, the airflow rate in the columns has to be adjusted to set DO in the ex situ columns within the range of DO in the examined aeration tank (ASCE 2018; Boyle 1983). This operation preserves steady-state conditions of DO and aims to reproduce the gas transfer found in the aeration tank as close as possible in the ex situ test column. In this case the setup resembles the in situ off-gas hood method, provided that the same diffuser type, diffuser density, and tank depth are implemented as in the examined aeration tank.

### *Sensitivity analysis of *ex situ* off-gas measurements*

ASCE 18–18 gives little information about measurement uncertainty of the off-gas method. It remains unclear which input quantity is most important to produce accurate results. The principle of sensitivity analysis (SA) is to identify the effect of changes of input quantities on the model output (i.e., the α-factor) (Turányi 1990). Examined input quantities to determine the α-factor as described above were off-gas oxygen (*O*_2,e_) and carbon dioxide concentrations (CO_2,e_), water temperature (*T*_w_), dissolved oxygen (DO and *C*_(t)_), electrical conductivity (EC) of the AS, atmospheric pressure (*p*_atm_), and the airflow rate (*q*_air_). In the underlying model to determine α-factors, some input quantities are correlated (especially *O*_2,e_, CO_2,e_, DO), e.g., higher CO_2,e_ values generally correlate with lower *O*_2,e_ values. The model is non-additive because input quantities interact with each other. This means that changing two inputs has a different effect on the output than the sum of their individual effects which must be considered in sensitivity analysis (Saltelli et al. 2004). Instead of simulating this dependency in the input quantities during the sampling process with individual models, we collected results of long-term measurements in a conventional activated sludge (CAS) WWTP with 700,000 population equivalent treating municipal wastewater over a period of 11 months. The resulting dataset contains 10,700 recorded α-factors as 1-h intervals. In this dataset distribution of input, quantities represent typical operation of a CAS plant including seasonal variations and therefore cover the range of input quantities required for a global sensitivity analysis (Saltelli et al. 2004; Sudret 2007).

Applying the methodology of sensitivity and uncertainty analysis, we examined the following aspects of α-factor determination with the ex situ off-gas method:

#### Method 1: Examine the individual influence of measured input quantities

An elementary “one factor at a time” (OAT) analysis only considers the relationship between the output and the variation of one individual input quantity around one baseline case where all other input quantities are kept at their nominal values (Saltelli 1999). This local method would be restricted to one observation of input quantities at a time (baseline case) to determine the α-factor. To consider the range of input quantities, we performed the analysis for our whole dataset and reported average deviations of the α-factor. The results were generated by varying all observations of a specific input quantity by ± 1.0% and ± 5.0% from their nominal values (baseline cases) and recalculating the average α-factor of the dataset. The baseline cases are the input quantities and corresponding α-factors as determined by ASCE 18–18 (2018) from our dataset. Relative percentage change of this value and the average α-factor of the dataset was calculated for comparison of variations of all input parameters. In elementary OAT, any interactions of input quantities are discounted. Nonetheless, this elementary OAT analysis can be performed if the variation of input quantities is small (Saltelli 1999; Saltelli et al. 2019). The small variation of ± 1.0% and ± 5.0% is chosen to represent typical measurement uncertainties of the input quantities.

#### Method 2: Examine the individual influence of correction factors

We applied the same method as in Method 1 and varied the correction factors θ and β as well as the conversion factor for TDS/EC according to their ranges found in literature.

#### Method 3: Estimate measurement uncertainty of our setup

We performed an uncertainty analysis to estimate the measurement uncertainty to expect when determining α-factors with our ex situ off-gas columns. The measurement uncertainty of the α-factor was affected by the measurement uncertainty of all instruments involved to measure input quantities. A common approach is to use a derivative based method for error propagation to determine a combined standard uncertainty (Joint Committee for Guides in Metrology 2008). However, this uncertainty would only be valid locally for an individual measurement and does not consider the distribution of errors. To take these aspects into account, we estimated the uncertainty for all measurements in our dataset by the following steps: To create a base for comparison, all observations of recorded input quantities and thereof determined α-factors in our dataset were regarded as “true” values, i.e., reference quantity values. Random measurement error of instruments was simulated by sampling 4000 values of every input quantity according to the instrument’s individual measurement uncertainty for every observation in our dataset (*n* = 10,700). A detailed overview of a priori instrument measurement uncertainties and their distributions which are specific to our pilot setup is listed in Appendix 2 of this paper and technical information of each instrument is also provided by manufacturers online. Most measurement uncertainties were chosen according to technical information by the manufacturer. However, because optical sensors for measurement of dissolved oxygen were operated in activated sludge the uncertainty of ± 1% of reading stated by manufacturer was considerably lower than our own measurements. Therefore, we assumed an uncertainty of ± 0.1 mg·L^−1^ (uniform distribution) ± 5% of reading (± SD, normal distribution) as described in Appendix 2. In total, 42 million theoretical α-factors were determined based on the instrument measurement uncertainty that represented the expected uncertainty of the α-factors defined as “true” values. Finally, theoretical α-factors were compared with the measured “true” α-factors in our dataset.

#### Method 4: Examine the individual influence of measured input quantities in our setup

Sobol’ sensitivity indices were determined in a global sensitivity analysis. The global SA estimated the output uncertainty due to the uncertainty of individual input quantities or combinations thereof. Sobol’ indices were calculated from a decomposition of the output’s variance. The aim was to identify the impact of input quantities on measurement uncertainty of α-factors for our specific pilot plant. As in Method 3, the results are based on the specific measurement uncertainties related to the instruments and sensors used in our pilot plant (see Appendix 2) and illustrate the importance of all input quantities’ measurement uncertainty when performing off-gas tests with the ex situ method. The general concept is described in Saltelli et al. (2004) and first introduced by Sobol’ (1993). We used a Monte Carlo estimation of Sobol’ indices with improved formulas of Jansen (1999) and Saltelli et al. (2010) to determine first-order and total effects Sobol’ sensitivity indices. A practical application of this SA can be found in Jadun et al. (2017), who compared variance-decomposition methods on a real model and evaluated it as most suitable to determine total effects indices.

Statistics and visualization were done using R 3.6.3 (R Core Team 2020), tidyverse package (v1.3.0) for visualization (Wickham et al. 2019), data.table package (v1.14.0) for data handling (Dowle and Srinivasan 2021) and sensitivity package (v1.26.1) to perform sensitivity analysis (Iooss et al. 2021).

## Results and discussion

First, we discuss the results of the sensitivity analysis to point out theoretical causes of measurement uncertainty. Afterwards, we present our results from a direct comparison of α-factors measured simultaneously in two pilot reactors from the same AS zone. Based on this, we discuss possible causes of random and systematic error affecting the ex situ off-gas method’s measurement uncertainty.

### OAT sensitivity analysis of α-factor determination

An average α-factor of 0.70 was calculated according to ASCE 18–18 for our whole dataset of measured input quantities. Table 1 displays the relative change from this average when all observations of an individual input quantity were adjusted by ± 1% or ± 5%, see Method 1. The mean value ± standard deviation (SD) of all input quantities is listed to characterize the dataset underlying the analysis.

**Table 1:** OAT sensitivity analysis: relative percentage change of mean α-factor for adjusted input parameters

Input parameter	Mean ± SD	α-factor: Rel. perc. change (%) calculated with input quantities adjusted by			
		− **5%**	− **1%**	** + 1%**	** + 5%**
O_2_ in off-gas (*O*_2,e_)	17.9 ± 0.7%	+ 31.2	+ 6.3	− 6.3	− 31.9
Water temperature (*T*_w_)	18.4 ± 2.9 °C	+ 1.8	+ 0.4	− 0.4	− 1.7
Atmospheric pressure (*p*_atm_)	1,013 ± 9 hPa	+ 1.3	+ 0.3	− 0.2	− 1.2
Dissolved oxygen (DO)	2.1 ± 1.4 mg·L^−1^	− 1.2	− 0.3	+ 0.3	+ 1.3
CO_2_ in off-gas (CO_2,e_)	2.2 ± 0.4%	+ 0.7	+ 0.1	− 0.1	− 0.7
Volume spec. airflow rate (*q*_air_)	1.5 ± 0.3 Nm^3^·m^−3^·h^−1^	+ 0.5	+ 0.1	− 0.1	− 0.5
Electrical conductivity (EC)	1380 ± 360 µS·cm^−1^	+ 0.1	0.0	0.0	− 0.1

The input quantities are sorted by descending absolute influence on the α-factor determination. When nominal values of O_2_ in off-gas were reduced by 5% across all measured observations, the mean α-factor increased by 31.2% based on the mean α-factor of 0.70. In contrast, a decrease of electrical conductivity by 5% increased α-factor negligibly by 0.1%. The exact relative percentage changes obtained by the analysis depend on the underlying dataset. The OAT sensitivity analysis confirms that the oxygen concentration in the off-gas is by far the most influential input quantity to determine the α-factor. Thus, maintenance and calibration of the gas analyzer is essential for off-gas testing. Adjusting water temperature, atmospheric pressure, and dissolved oxygen by up to ± 5% had similar impacts on the average α-factor. This theoretical approach ignores the fact that each sensor recording these input quantities has a different measurement uncertainty. Errors of more than ± 5% are common for airflow meters or DO sensors when used in AS. Additionally, the closer water temperatures were to 20 °C the lower the relative percentage change of α-factor, because of its standardization to 20 °C.

In Method 2, the same approach is applied to analyze the impact of correction factors for standardization. Inexact values of theses constants could be an additional source of measurement uncertainty (Joint Committee for Guides in Metrology 2008). Table 2 lists the relative or absolute percentage changes from the average α-factor of the dataset for variations of three standardization correction factors as input quantities.

**Table 2:** OAT SA: relative/absolute percentage change of mean α-factor for adjusted standardization factor

**Standardization factor**		**Adjustments of nominal value (** −**)** **Rel./abs. change of α-factors (%)**				
θ temperature correction factor (theta)	Input ( −)	1.008	1.02	1.024	1.028	1.047
	Abs. perc. change of α-factors (%)*	4.2	1.1	−	1.1	6.3
TDS/EC conversion factor	Input ( −)	0.55	0.62	0.67	0.72	0.90
	Rel. perc. change of α-factors (%)	+ 0.2	+ 0.1	−	− 0.1	− 0.3
β-factor (beta)	Input ( −)	0.9	0.95	0.991	0.994	0.998
	Rel. perc. change of α-factors (%)	− 9.2	− 4.1	−	+ 0.3	+ 0.7

^*^Absolute percentage change of α-factor was determined for variations of Θ because deviations changed from positive to negative (and vice versa) at water temperatures of 20 °C

The temperature correction factor θ applies a geometric correction to standardize mass transfer of oxygen to 20 °C. It is set to 1.024, but the empirically determined factor attempts to combine several effects such as changes in diffusivity of oxygen, viscosity, or surface tension. Reported values range from 1.008 to 1.047 (Stenstrom and Gilbert 1981), while ranges from 1.020 to 1.028 are reasonable according to US Environmental Protection Agency (1989). As theta is influenced by turbulence, it depends on the type of aeration system. Changing theta to a different factor requires support of substantial data (Stenstrom and Gilbert 1981). Within the range of 1.024 ± 0.004, the average α-factor of our dataset deviated by 1.1%. However, temperature correction becomes more influential for off-gas measurements at more extreme temperatures than present in our dataset (18.4 ± 2.9 °C).

The conversion from total dissolved solids to electrical conductivity is 2 mg·L^−1^ TDS = 3 µS·cm^−1^ (DWA 2007), see Appendix 1, equation A5. The factor 0.67 is confirmed by Behnisch et al. (2021) who determined an average of 0.7 for various salts. AWWA standard methods list a broader range between 0.55 and 0.9 (AWWA, 2017). However, the effect on α-factor determination is negligible (below ± 0.3%) at the salt concentrations expected in municipal wastewater. In this case, an adjustment of the β-factor also has low impact on the resulting α-factors. In our dataset, a mean β-factor of 0.991 was determined by equation A5. Uncertainty about the correct estimate of the β-factor in municipal wastewater remains as Eckenfelder et al. (1956) report β as approximately 0.95 and ASCE 18–18 states that it can vary from 0.8 to 1.0, but is generally close to 1.0. An adjustment of β to 0.95 or 0.9 results in a relative change of α-factor of − 4.1% and − 9.2%, respectively. If equation A5 did not consider the effect of salts on effective oxygen saturation concentration correctly, it would directly impact the α-factor. For certain industrial (and possibly municipal) wastewaters, this could introduce a systematic error when determining the α-factor.

### *Variance-based sensitivity analysis of the *ex situ* off-gas method*

**Fig. 2:**
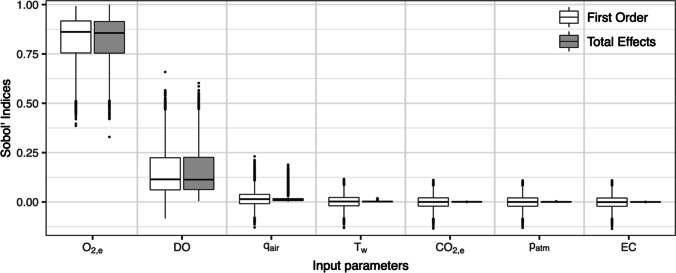
Comparison of first-order and total effects Sobol’ sensitivity indices for input quantities to determine α-factors: oxygen in off-gas (*O*_2,e_), dissolved oxygen (DO), airflow rate (*q*_air_), water temperature (*T*_W_), carbon dioxide in off-gas (CO_2,e_), atmospheric pressure (*p*_atm_), and electrical conductivity (EC)

The OAT sensitivity analysis described before did not consider possible interactions of input quantities on the α-factor and only selectively compared importance of input quantities for fixed variations of ± 1% and ± 5%. Sobol’ indices based on variance decomposition detected interactions of input quantities and considered their differing measurement uncertainties (see Method 4). The resultant first-order and total effects Sobol’ sensitivity indices for all input quantities are sorted in descending importance from left to right in Fig. 2. Boxplots visualize the distribution of indices for all 10.500 samples of the underlying dataset instead of adding bootstrap confidence intervals for every index.

Oxygen concentration in off-gas (*O*_2,e_) has the highest first-order Sobol’ index followed by dissolved oxygen (DO). These Sobol’ indices show the influence of each input quantities’ measurement uncertainty on the variance of the output (i.e., the α-factor). Compared with the previous OAT sensitivity analysis (see Table 1), this confirms the importance of oxygen concentration in the off-gas whereas the impact of dissolved oxygen is higher than before. Hence, we can conclude that accurate measurement of oxygen in off-gas and dissolved oxygen must be prioritized for reliable off-gas testing with the instruments used in our pilot setup. In contrast, all other input quantities have low first-order and total effects indices which means that their measurement uncertainty had a negligible effect on the uncertainty of the α-factor.

The output was primarily influenced by first-order effects because these were equal to total effects. Although the model is non-additive, no significant interactions were present when sampling input quantities within their measurement uncertainty. Otherwise, total effects indices would be larger than first-order indices. Interactions were present in the model although larger deviations of input quantities were tested (data not shown). On average, sum of first-order and total effects Sobol’ indices were close to 1, which also confirms that interactions between input quantities were negligible. Some first-order Sobol’ indices were negative for the less influential input quantities. This was not caused by correlated input quantities because sampling was random. Negative first-order Sobol’ indices can occur when the sample size is insufficient (Glen and Isaacs 2012) or when output is not distributed normally (Menberg et al. 2016). Nonetheless, indices can be assumed zero because they were distributed evenly around zero. Although the first-order indices could be not as robust as under perfect conditions, they still demonstrate a distinct difference in the input quantities’ importance as discussed above.

### *Random measurement error of *ex situ* off-gas tests*

We simulated random measurement error of our setup based on the input quantities’ individual measurement uncertainty as described in Method 3. The resulting difference of the “true” α-factors and sampled α-factors produce random measurement errors to estimate the measurement uncertainty across a dataset of long-term measurements. The average α-factor and its standard deviation was 0.70 ± 0.025 (relative standard deviation of ± 3.7%).

**Fig. 3:**
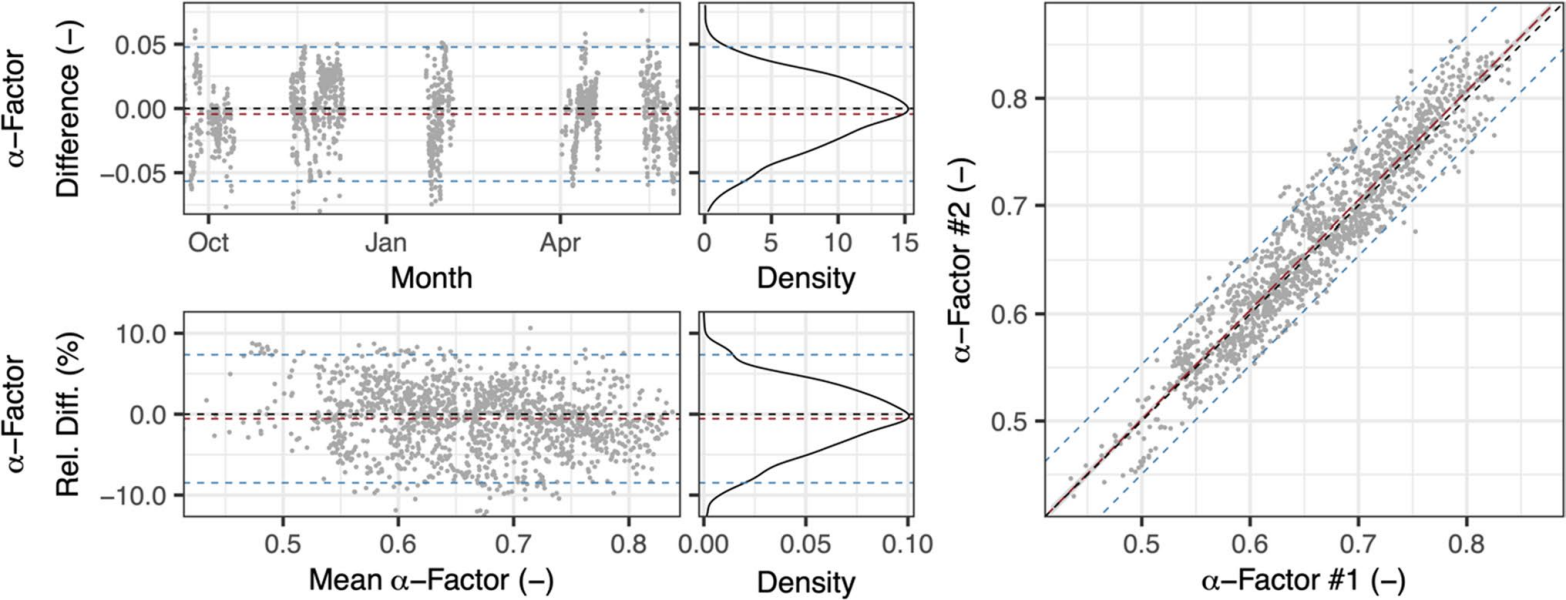
Comparison of α-factors simultaneously determined in two ex situ off-gas columns

Next, we estimated the measurement uncertainty of our setup by considering measurement error from comparison measurements. Our dataset included periods where both pilot reactors were operated at the same airflow rate and hydraulic retention time while transferring AS from the same aeration zone. In total, 1400 pairs of simultaneously determined α-factors collected at 1-h intervals provided a direct comparison to estimate the pilot setup’s measurement uncertainty. This direct comparison of two identically equipped and operated ex situ off-gas columns was a suitable method to estimate the method’s measurement uncertainty because no activated sludge with a known α-factor can be used for calibration. The results are shown in Fig. 3.

Figure 3 is divided into five separate diagrams. The upper diagrams display the difference of α-factor #1 and α-factor #2 over a long-term measurement period (left) and their resulting distribution (center). The lower counterparts show the relative difference (ratio of difference and common mean of both α-factors) for the mean α-factor of both setups (left) and the resulting distribution (center). The right diagram directly compares each pair of α-factors. Dashed lines represent the ideal case without any difference (black), the average of all observations of our dataset (red) and ± 2 SD around the mean or the corresponding 95%-prediction interval (blue). This comparison of two pilot setups shows that individual measurements of α-factors approximately follow a normal distribution with a SD of ± 4% of relative difference, whereas long-term testing provides more consistent results. The average of all observations (red dashed line) is close to the ideal case (black dashed line) with a relative difference lower than 1%.

The measurement error equals random measurement error plus systematic measurement error. As discussed in the next section, systematic measurement errors could not be excluded or corrected as the offset shifted between measurement periods. Figure 3 thus visualizes spread and distribution of random and systematic error values. Based on these the measurement uncertainty of the individual setups was estimated. One assumption therefore is the normal distribution of the observed differences of α-factors (compare Fig. 3, center top) and the differences of α-factor #1 and #2 with the ideal case. This assumption is common for random error of measurements. If the distribution is the same for both setups their mean (*µ*) and standard deviation (*σ*) can be derived from:

3 $$\mathcal{N}\left({\mu }_{obs},{\sigma }_{obs}^{2}\right)=\mathcal{N}\left({\mu }_{1}, {\sigma }_{1}^{2}\right)-\mathcal{N}\left({\mu }_{2}, {\sigma }_{2}^{2}\right)$$

with:

*N*(μ_obs_, σ_obs_^2^): Normal distribution of the observed (measured) differences of α-factors as shown in Figure 3 with mean *μ*_obs_ = *μ*_1_ − *μ*_2_ = −0.0044 ≈ 0 and standard deviation *σ*_obs_ = 0.026
*N*(μ_1,2_, σ_1,2_^2^): Normal distribution of the individual setups determining α-factor #1 and #2.

Because standard deviations for the individual setups (*σ*_1_ and *σ*_2_) were assumed identical they can be calculated as follows:

4 $${\sigma }_{\mathrm{obs}}^{2}={\sigma }_{1}^{2}+{\sigma }_{2}^{2}=2{\sigma }_{\mathrm{1,2}}^{2}$$

5 $${\sigma }_{\mathrm{1,2}}=\sqrt{\frac{{\sigma }_{\mathrm{obs}}^{2}}{2}}=0.018$$

While the difference of means *μ*_1_ and *μ*_2_ was close to zero, the standard deviations *σ*_1_ and *σ*_2_ were calculated from Eq. (5) as 0.018 for α-factors in the dataset of the comparison. At an average α-factor of 0.66 in the dataset, the mean relative standard deviation was ± 2.8%. The lower left diagram in Fig. 3 shows that relative difference of α-factors does not change significantly at lower or higher α-factors, which indicates that the relative standard deviation can estimate measurement uncertainty across the whole range of possible α-factors. Table 3 compares these measurement error results of measured values from two pilot setups with the results simulated with the uncertainty analysis (see Method 3).

**Table 3:** Comparison of measurement uncertainty based on simulation and measurements of ex situ off-gas tests

Data source and analysis	Simulated in uncertainty analysis	Parallel measurement in two ex situ pilot reactors
Number of observations in dataset	*n* = 10.500	*n* = 1.400 (in each reactor)
Mean α-factor of dataset	0.70	0.66
Mean standard deviation ( −)	± 0.025	± 0.018
Mean relative standard deviation (%)	± 3.7	± 2.8

The average values of standard deviation and relative standard deviation are similar for both approaches. However, data analyzed from parallel measurement in two ex situ pilot reactors was compressed to 1-h intervals. In contrast, the values simulated in the uncertainty analysis represented the random error of measurement expected for instrument readings within the overall response time of all instruments. This period is shorter than one hour and cannot be determined exactly for our setup because response time of instruments varied, and gas sampling was dependent on airflow rate. The values simulated in the uncertainty analysis overestimate uncertainty, because more than one distinct measurement could take place within 1 h and thereby decrease the overall random measurement error.

Repeating off-gas measurements is generally recommendable due to random measurement errors. Our results show that a considerable measurement error could remain if only a single 1-h interval of an α-factor was determined. Repeated long-term measurements can compensate for this as the following example illustrates: In the case of our pilot reactors, a single measurement of an α-factor of 0.66 would be associated with a relative standard deviation of ± 2.8%. Repeating this measurement 5, 10, or 20 times would decrease relative standard deviation to 1.3%, 0.9%, or 0.6%, respectively. This example is valid under the assumption of a pure random measurement error. However, a systematic measurement error influencing multiple observations in sequence could still have a larger impact than demonstrated here.

### *Systematic measurement error of *ex situ* off-gas tests*

**Fig. 4:**
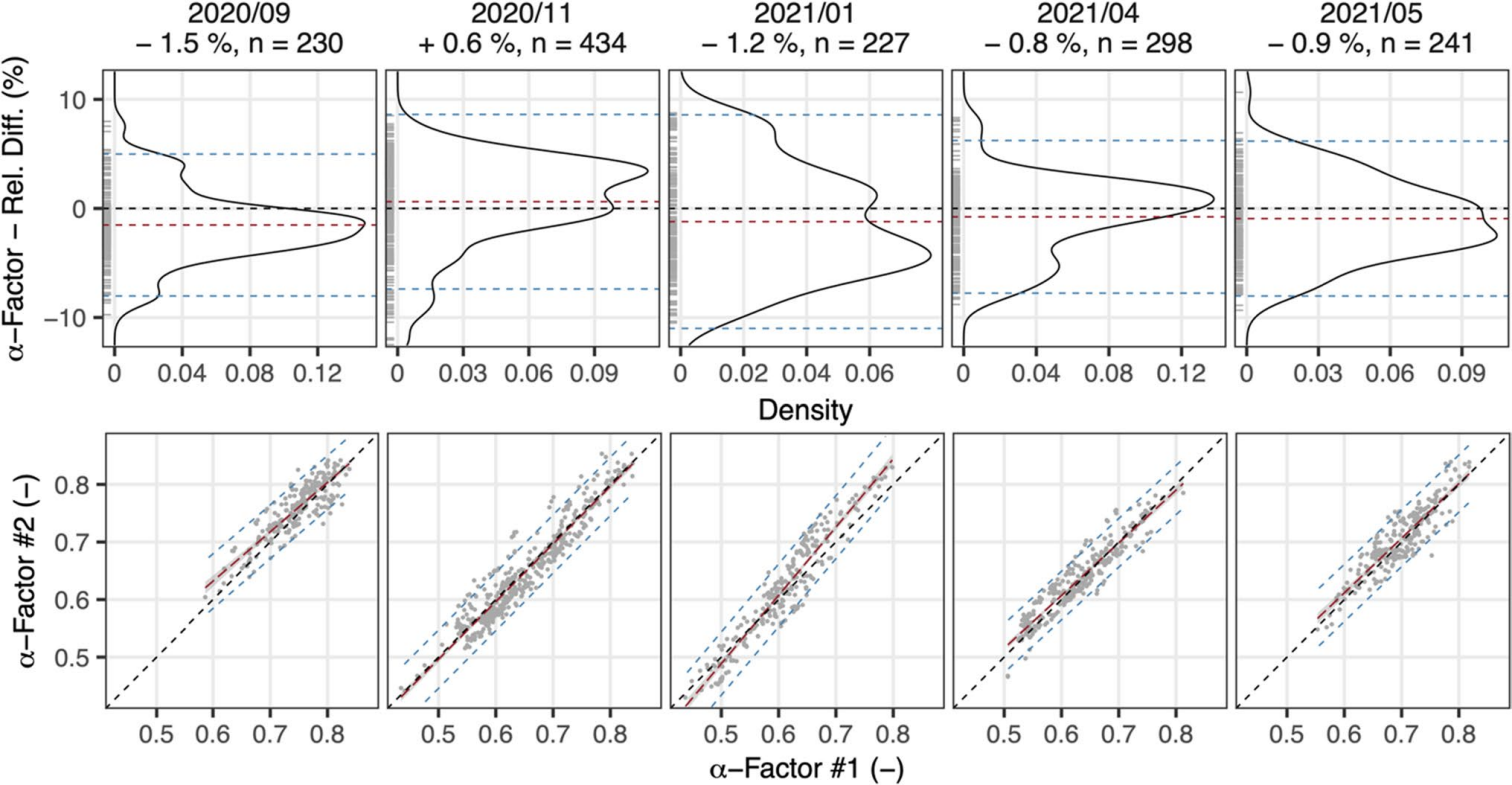
α-factor comparison split into five off-gas measurement periods

Comparative measurements were performed in five distinct periods of more than 10 days that are separated in Fig. 4. The titles of the individual diagrams state the period of measurement, average relative difference (%), and the number of recorded 1-h intervals (*n*) within that period (maintenance excluded). Diagrams on top present the distribution of relative difference with a rug marking individual datapoints while diagrams on the bottom show the direct comparison of each pair of α-factors. As in Fig. 2, dashed lines indicate the ideal case of no deviation (black) the mean within that period (red) and ± 2 SD around the mean or the 95%-prediction interval (blue).

The comparison shows that the distribution of relative differences and their average varied between measurement periods. Relative differences were not always normally distributed. This suggests that the ex situ off-gas measurement was subject to systematic measurement errors that changed between or within measurement periods. The systematic measurement error as a relative difference was within a range lower than ± 1.5% in our setup. It is worth mentioning that this cannot identify systematic measurement errors occurring at the same time and evenly in both setups. Therefore, a systematic measurement error could be higher than the reported relative difference of ± 1.5%.

Systematic measurement error could be caused, among other reasons, by fouling of diffusers, biofilm growth, sensor drift, or imperfect clean water testing and therefore reduced by proper maintenance of the setup and extensive clean water testing. Based on our data, systematic measurement error could not be quantitatively attributed to potential causes as discussed below. Consequently, an unknown systematic measurement error cannot be corrected when estimating the measurement uncertainty. The comparison in Fig. 4 and thereof derived relative difference of up to ± 1.5% could not conclusively distinguish random measurement error from systematic measurement error. Nonetheless, it exemplarily demonstrates the effect and acknowledging the potential causes listed below may help to minimize systematic measurement errors when performing ex situ off-gas measurements.

Fouling, scaling, and aging of diffusers affects the oxygen transfer performance of an aeration system. Odize et al. (2017) found that reverse flexing helped to reduce pressure loss during operation but did not improve fouling factor effectively. Therefore, the membrane surface of diffusers was cleaned with high pressure before the individual measurement periods to mitigate fouling. Within the long-term off-gas testing period of 11 months, pressure loss increased on average by 2 kPa for both pilot reactors, but pressure loss and relative deviations of α-factors were not correlated.

Preventing excessive biofilm growth within the reactors is critical. Sessile biomass in the ex situ columns increases overall oxygen respiration and therefore alters DO concentrations and oxygen driving force in the columns when compared to suspended biomass in the AS tank. Consequently, ex situ columns could fail to accurately measure oxygen transfer conditions in an AS tank because of this systematic error. Reactor tank walls were cleaned regularly concurrently with diffusers to prevent this effect. Overall, no significant biofilm production was observed during testing. However, the impact of biofilm growth remains an unquantifiable source of error.

Sensor drift of off-gas analyzer or DO sensors could result in a systematic measurement error. A small drift of oxygen concentrations in the off-gas would have a disproportionate effect on the α-factor as shown by the sensitivity analysis. Regular calibration depending on the gas analyzer’s requirement is advisable. Outliers in collected data could be identified a posteriori by large drifts marked in a calibration protocol. Moreover, biofilm growth on DO sensors submerged in AS affected their accuracy and required regular cleaning. A duplicate or triplicate measurement is advisable as it allows to identify outliers of single defective sensors a posteriori. Once these outliers were detected and removed from our dataset, no correlation with the relative difference of the α-factor was apparent. From our experience, other sensors and instruments involved in the measurement were less error-prone. Details on implementations in our setup are stated in section the “Methods” section. 

Clean water testing results are the denominator of the α-factor. Results of linear regression equations (SOTR ~ *q*_air_) were similar for both reactors, but deviations were more probable at extreme airflow rates. Extensive clean water testing beyond the usual range of set airflow rates is advisable. Nonetheless, at airflow rates below 0.5 Nm^3^·m^−3^·h^−1^ accuracy of the airflow meter was insufficient in our setup. Once α-factors determined at low airflow rates were excluded, no correlation with the relative deviation between pilot reactors was apparent.

Water volume directly affects determination of oxygen transfer parameters and should be kept constant during testing as described in the “Methods” section. In our setup, no deviation related to differences of tank volume was expected because of the identical geometry of aeration columns.

### Limitations of the ex situ off-gas method

Unlike in situ measurements with off-gas hoods at the surface of an aeration tank, oxygen transfer in the AS is examined ex situ with the method discussed here. Sludge transfer from an AS tank and aeration in a column with a different geometry than the AS tank could skew the α-factors determined with ex situ off-gas measurements under certain conditions.

Firstly, the positioning of sludge transfer hoses in the AS tank limits the zone that can be examined with the ex situ columns. In situ off-gas hoods are similarly restricted to cover small areas of an aeration tank. In the case of insufficient mixing in the AS tank, sludge characteristics at the sampling point could result in an undetected error. Therefore, sludge transfer hoses should be positioned in a sufficiently mixed zone.

Secondly, during transfer of aerated AS in sludge transfer hoses additional oxygen is dissolved from the gas phase while oxygen consumption of the biomass reduces it. In our dataset, oxygen transfer rates in the ex situ columns were on average 71 ± 16 g·m^−3^·h^−1^ and oxygen uptake rates were similar at 68 ± 17 g·m^−3^·h^−1^. However, it remains unclear whether the two opposing effects were balanced in the sludge transfer hoses. It is possible that more oxygen is dissolved than consumed under turbulent flow conditions in the hoses, which would result in an overestimation of the α-factor. Therefore, hose lengths should either be as short as possible or of the same length to reduce a potential systematic measurement error. In our application, hose lengths of up to 100 m were used.

**Fig. 5:**
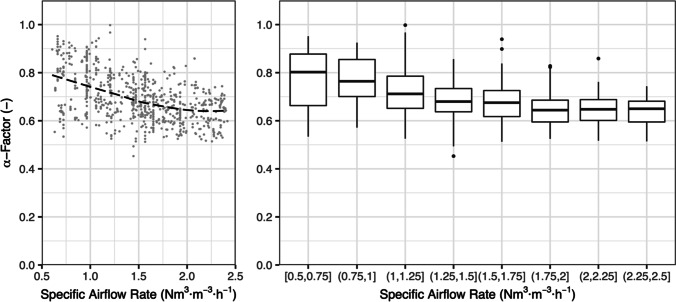
α-Factors at specific airflow rates in the ex situ column at constant sludge inflow

Thirdly, the sludge transfer into the column produces a lateral flow at the height of the diffusers that is only present during off-gas testing, not during clean water testing. Figure 5 shows the relationship of α-factor and volume specific airflow rate for our setup where off-gas testing was performed during dry weather in the same aeration zone of a CAS WWTP. Off-gas tests were performed at a constant sludge inflow with a HRT of 15 min so that turbulence in the columns was only influenced by airflow rate. The same data is depicted as individual data points with a local polynomial regression fit as a dashed line (left) and boxplots (right).

Both diagrams show that α-factor increases at lower airflow rates. During off-gas testing in AS, oxygen transfer is improved by higher turbulence as the rising bubble plume is additionally mixed by the sludge inflow. Consequently, a systematic overestimation of α-factors is possible, especially at low airflow rates where gas–liquid ratio is particularly low. The effect can be reduced by setting higher airflow rates that create a similarly high turbulence in off-gas and clean water testing. Nonetheless, this systematic error is setup specific and should be quantified for each ex situ column. Although Fig. 5 suggests that a further decrease of α-factor is limited at high airflow rates, the “true” α-factor in the AS tank is difficult to determine with the ex situ method. Nonetheless, an off-gas measurement with in situ off-gas hoods is preferable if α-factors are determined to design the aeration system of the examined AS tank.

Fourthly, determination of standard aeration efficiency (SAE) relies on accurate measurement of power consumption of blowers. Blowers equipped in a pilot-scale ex situ setup cannot accurately represent the power consumption of aeration in a full-scale AS tank. In contrast, in situ off-gas measurements with off-gas hoods use the blowers of the AS tank and should therefore be preferred to determine SAE.

## Conclusions

Below, we summarize our findings about the application of ex situ column off-gas testing and its measurement uncertainty to determine α-factors in activated sludge tanks.

1. We determined the most important input quantities of the ex situ off-gas method with a “one factor at a time” (OAT) sensitivity analysis and a global variance-based sensitivity analysis using Sobol' indices. The analysis was based on measurement uncertainties of required instruments and revealed that oxygen concentration in off-gas was the most important input quantity to determine oxygen transfer parameters (e.g., the α-factor). It was followed by dissolved oxygen concentration because its measurement in activated sludge could be unreliable. The uncertainties of all other input quantities were negligible.
2. We performed an uncertainty analysis for a dataset of long-term measurements based on the measurement uncertainties of instruments in our pilot setup and estimated measurement uncertainty of the α-factor as a relative standard deviation of about ± 3.7%. A direct comparison of α-factors from parallel operation of ex situ pilot reactors under the same conditions transferring AS from the same aeration zone resulted in a similar relative standard deviation of about ± 2.8%. This value represents the measurement uncertainty of a single value recorded with the ex situ off-gas method. The theoretically determined relative standard deviation of ± 3.7% and the relative standard deviation of ± 2.8% determined from practice in our pilot setup are lower than a measurement uncertainty of ± 5 to 10% estimated in literature before. Thus, a more accurate off-gas measurement seems possible. We recommend estimating the measurement uncertainty of α-factors theoretically for the installed instruments when planning an ex situ pilot setup as shown in *Method 3*. In any case, repeating measurements is advisable to produce more accurate results and reporting a measurement uncertainty of the method is beneficial to interpret results. Nonetheless, systematic measurement errors can be present and caused, e.g., by fouling of diffusers, biofilm growth, sensor drift, or imperfect clean water testing. In our experience, systematic measurement errors of about ± 1.5% of α-factor can be caused by these issues which can rarely be identified a posteriori and only reduced by proper maintenance of the setup.
3. The α-factor is standardized with correction factors to consider the influence of temperature and total dissolved solids on oxygen transfer according to standard guidelines. OAT sensitivity analysis revealed that impact of correction factors on the α-factor was lower than measurement uncertainty of the most important input quantities (oxygen concentration in off-gas and activated sludge). However, temperature correction factor θ became increasingly important when off-gas testing was conducted in activated sludge at water temperatures deviating from 20 °C. Because θ was empirically estimated as 1.024, an unknown systematic measurement error could result when comparing oxygen transfer results from tests at significantly different temperatures. The influence of salts on the effective saturation concentration as represented by the β-factor was estimated with a formula that has negligible effect on α-factor. Nonetheless, for off-gas tests in AS treating industrial wastewater with high salt contents the β-factor should be validated by additional tests to avoid a systematic measurement error.
4. In general, the findings for the ex situ off-gas method are transferable to in situ off-gas hoods because the same instruments are used to determine the α-factor. We outlined systematic influences that differentiate the methods from each other, such as changes of oxygen balance in inflow or higher turbulence in ex situ columns due to sludge transfer. We conclude that the ex situ method is not suitable to determine α-factors to design aeration systems because a systematic overestimation of α-factor at low airflow rates is probable. In contrast, off-gas hoods are suitable to monitor oxygen transfer in activated sludge tanks, e.g., for compliance testing, because resulting α-factors represent in situ conditions. In addition, full coverage of tanks is less expensive and operation easier to maintain than with ex situ reactors. However, the possibility to operate ex situ reactors independently from AS tanks offers unique possibilities for research of oxygen transfer dynamics in AS and development of aeration equipment. It could see a future application in the parallel measurement of oxygen transfer and greenhouse gas emissions (such as nitrous oxide) in aerated and non-aerated zones.

## Data Availability

Not applicable.

## Appendix 1 Equations to determine the α-factor from measured input quantities

This appendix contains all equations used to determine the α-factor with the ex situ method. Most notable are the adjustments made to allow a determination of the α-factor based on online sensor data (estimating TDS from electrical conductivity and calculating *C*_S,T,St_ from a polynomial). With these a reader can replicate the sensitivity analysis discussed above with own data.

**MR**_**i**_**: Molar ratio of oxygen to inert substances**

A1 $$M{\mathrm{R}}_{\mathrm{i}}=\frac{\frac{{O}_{2,\mathrm{in}}}{1-{\mathrm{CO}}_{2,\mathrm{in}}}}{1-\frac{{O}_{2,\mathrm{in}}}{1-{\mathrm{CO}}_{2,\mathrm{in}}}}=0.265$$

with:

- *O*_2,in_: Inlet oxygen concentration of 20.946 %
- CO_2,in_: Inlet carbon dioxide concentration of 0.0407 %

***C***_**S,T,St**_**: Oxygen saturation concentration at water temperature *****T***_**w**_** (mg·L**^**−1**^**)**

A2 $${C}_{S,T,St}=\frac{2234.34}{{(T}_{w}+{45.93)}^{1.31403}}$$

with:

- *T*_w_: Water temperature (°C)

ASCE 18–18 refers to tabulated values by Benson and Krause Jr (1984), the polynomial above is defined in DWA-M 229–1 and calculates these values (DWA 2017).

***C***_**S,md**_**: Oxygen saturation concentration at mid-depth and standard conditions (mg·L**^**−1**^**)**

A3 $${C}_{S,md}=9.09\cdot \frac{{h}_{D}}{2\cdot 10.35}$$

with:

- *h*_D_: Blow-in depth (m)

Mid-depth saturation model based on DWA-M 209 (DWA 2007), the mid-depth model is also recommended in Jiang and Stenstrom (2012). The effective saturation depth is setup-specific and was about 50% of blow-in depth in our setup, which was determined in clean water tests by comparison with oxygen saturation at the surface according to DWA-M 209 (2007).

**OTE**_**f**_**: Oxygen transfer efficiency under process conditions (%)**

A4 $${\mathrm{OTE}}_{\mathrm{f}}=\frac{{\mathrm{MR}}_{\mathrm{i}}-\left(\frac{{O}_{2,e}}{1-{O}_{2,e}-C{O}_{2,e}}\right)}{{\mathrm{MR}}_{\mathrm{i}}}$$

with:

- *O*_2,e_: Oxygen concentration in off-gas (%)
- CO_2,e_: Carbon dioxide concentration in off-gas (%)

The parameter is calculated depending on the gas analyzer output and/or off-gas conditioning. For other variants the reader is referred to ASCE 18–18 (2018) or DWA-M 209 (2007).

***β*** **: β-factor (beta) (−)**

the ratio of oxygen saturation in process water to clean water at equivalent conditions of water temperature partial pressure.

A5 $$\begin{array}{ccc}\beta =1.00-0.01\cdot \frac{TDS}{1000}& \mathrm{or}& \beta =1.00-0.01\cdot \frac{\frac{2}{3}\cdot EC}{1000}\end{array}$$

with:

- TDS: Total dissolved solids (mg·L^**−**1^)
- EC: Electrical conductivity (µS·cm^**−**1^)

Online monitoring requires continuous measurement of TDS. It is therefore approximated with the electrical conductivity. A common conversion is 2000 mg·L^**−**1^ TDS = 3000 µS·cm^**−**1^ (DWA 2007).

***C***^*****^_**20**_**: Standardized effective oxygen saturation at process conditions (mg·L**^**−1**^**)**

A6 $${C}_{20}^{*}={{C}_{S,md}\cdot \tau \cdot \Omega =C}_{S,md}\cdot \frac{{C}_{S,T,St}}{9.09}\cdot \frac{{p}_{atm}}{101.325}$$

with:

- *τ*: temperature correction (tau) of effective saturation concentration (**−**)
- *Ω*: pressure correction (omega) of effective saturation concentration (**−**)
- *p*_atm_:atmospheric pressure (kPa)

**OTE**_**sp,20**_**: Oxygen transfer efficiency per unit of driving force at std. conditions (%/mg·L**^**−1**^**)**

A7 $${\mathrm{OTE}}_{\mathrm{sp},20}=\frac{OT{E}_{f}}{{C}_{20}^{*}-{C}_{(t)}}\cdot {\theta }^{20-{T}_{w}}$$

with:

- *C*_(t)_: Dissolved oxygen concentration in the ex situ column (mg·L^**−**1^)
- *θ*: Temperature correction factor (theta) = 1.024 (**−**)

**SOTE**_**pw**_ **: Standard oxygen transfer efficiency under process conditions (%)**

A8 $${\mathrm{SOTE}}_{\mathrm{pw}}=OT{E}_{sp20}\cdot {C}_{20}^{*}\cdot \beta$$

**SOTR**_**pw**_**: Standard oxygen transfer rate in process water (g·h**^**−1**^**)**

A9 $${\mathrm{SOTR}}_{\mathrm{pw}}={q}_{\mathrm{air}}\cdot 299.3\cdot {\mathrm{SOTE}}_{\mathrm{pw}}$$

with:

- *q*_air_: Airflow rate, e.g., volume specific (Nm^3^·m^**−**3^·h^**−**1^)

***α*** **: α-factor (alpha) (-)**

Ratio of *k*_L_a_pw_ in process water to *k*_L_a_cw_ in clean water at equivalent conditions of tank geometry, mixing, etc.

A10 $$\alpha =\frac{{\mathrm{SOTR}}_{\mathrm{pw}}}{{\mathrm{SOTR}}_{\mathrm{cw}}}$$

with:

- SOTR_cw_: standard oxygen transfer rate in clean water (g·h^**−**1^) measured at the same airflow rate q_air_as in process water, see also ASCE 2-06 (2007), EN 12255-15 (2004) or DWA-M 209 (2007). It is linearly dependent on the airflow rate and can therefore be calculated from a setup-specific linear regression model

## Appendix 2 Measurement uncertainty of input quantities to determine α-factors

Below, the measurement uncertainty of all sensors and instruments is listed that were used in the uncertainty analysis and sensitivity analysis. If not specified otherwise a normal distribution is assumed with a coverage factor of 1 (± 1 SD).

## ***O***_2,e_—oxygen concentration in off-gas

Paramagnetic sensor X-STREAM Enhanced, XEGP-C-14-B40-0-B40-0-O26-0-O26-0–000-0–3-0–8-0–0-1–0-0-B-B (Emerson Electric Co., MO, USA)

 ± 0.5% of reading (repeatability, confirmed by own measurements at ambient air).

## CO_2,e_—carbon dioxide concentration in off-gas

Nondispersive infrared sensor X-STREAM Enhanced, XEGP-C-14-B40-0-B40-0-O26-0-O26-0–000-0–3-0–8-0–0-1–0-0-B-B (Emerson Electric Co., Missouri, USA)

 ± 0.5% of upper range limit of 5% (repeatability, confirmed by own measurements in ambient air).

## DO—dissolved oxygen ***C***_(t)_

Digital, optical measurement of dissolved oxygen based on fluorescence quenching COS61D-1009/0-AAA1A4 (Endress + Hauser AG, Reinach, Switzerland)

According to technical information by the manufacturer measurement uncertainty is stated as 0.01 mg·L^−1^ or ± 1% of reading. However, this accuracy recorded under laboratory calibration conditions is far from the readings we measured in activated sludge. Even under laboratory conditions Helm et al. (2018) found a drift of ± 0.2 mg·L^−1^ of optical sensors within 1 month of use. Näykki et al. (2013) conservatively estimated uncertainty of measurement at ± 0.15 mg·L^−1^ (± SD) for reference DO measurements in interlaboratory comparison for several types of DO sensors. Based on own measurements with two or three sensors operated in the same reactor column (though at different depth of submergence), we defined a reasonable measurement uncertainty of optical DO sensors in activated sludge as ± 0.1 mg·L^−1^ (uniform distribution) ± 5% of reading (± SD, normal distribution). This is a conservative estimate because the mean value of two or three sensors is used to determine the α-factor.

## ***T***_w_—water temperature

Temperature probe in digital dissolved oxygen sensor based on fluorescence quenching COS61D-1009/0-AAA1A4 (Endress + Hauser AG, Reinach, Switzerland)

 ± 0.75% of reading (uniform distribution, based on own measurements with two or three sensors operated in the same reactor column, no information provided by manufacturer).

## EC—electrical conductivity in activated sludge

Toroidal potentiometric conductivity sensor Indumax CLS50D -10M7/0-AA1B31 (Endress + Hauser AG, Reinach, Switzerland)

 ± (5 µS·cm^−1^ + 0.5% of reading) (maximum measured error, a uniform distribution is applied for sampling).

## ***p***_atm_—atmospheric pressure at blower air intake

Absolute and gauge pressure Cerabar PMC21-21W0/0-AAl U2KBWBJA (Endress + Hauser AG, Reinach, Switzerland)

 ± 0.3% of upper range limit of 2 bar (maximum measured error, a uniform distribution is applied for sampling).

## ***q***_air_—airflow rate

Thermal mass flow meter t-mass A 150-14D9/0-6AAB15 (Endress + Hauser AG, Reinach, Switzerland)

 ± 4% of reading (uncertainty increases at airflow rates below 6 Nm^3^·h^−1^ that were not set).
